# High MSWDs are not the problem, low ones are

**DOI:** 10.1093/nsr/nwaf036

**Published:** 2025-02-07

**Authors:** Pieter Vermeesch

**Affiliations:** Department of Earth Sciences, University College London, UK

The mean square weighted deviation (MSWD) is a widely used measure of statistical dispersion in geochronology. It quantifies the degree to which analytical uncertainty explains the observed scatter of some data around a weighted mean or regression line [1]. There is a misconception among some geochronologists that low MSWD values are ‘good’ and that high MSWD values are ‘bad’. This attitude is misguided.

- Overdispersed datasets are not the exception but the rule in geochronology.
- The absence of excess dispersion in geochronological datasets should raise suspicion, not praise.
- Dispersion should not be feared but quantified.

To illustrate these points, consider a collection of *n* igneous zircon crystals, and let $\lbrace t_1,\ldots ,{t_i},\ldots ,t_n\rbrace$ be their true ages. Compare the following two scenarios.

Scenario 1. The zircons all formed at exactly the same time *t*, so that $t_i=t$ for all $1\le {i}\le {n}$.

Scenario 2. The zircons formed over a finite time interval, so that ${t_i}\ne {t_j}$ if ${i}\ne {j}$.

The true ages $t_i$ are unknown, but can be estimated from isotopic data. Let $\hat{t}_i$ be the measured *date* of the *i*th zircon crystal, and let $\sigma _i$ be its known analytical uncertainty. Assume that $\hat{t}_i$ was drawn from a normal distribution with mean $t_i$ and standard deviation $\sigma _i$.

Under Scenario 1, the true zircon formation age *t* can be estimated (as $\bar{t}$) by taking the weighted mean of the measured dates:


(1) \begin{eqnarray*}
\bar{t} = \frac{\sum _{i=1}^n\hat{t}_i/\sigma _i^2}{\sum _{i=1}^n1/\sigma _i^2}.
\end{eqnarray*}

The weighted mean can be misleading under Scenario 2. For example, if the true ages follow a bimodal distribution then the weighted mean date would fall between the two modes. It is, therefore, of considerable interest to differentiate between Scenarios 1 and 2. The MSWD offers a way to do this. The MSWD of the weighted mean is defined as


(2) \begin{eqnarray*}
\mbox{MSWD} = \frac{1}{d\!f}\sum _{i=1}^n\bigg (\frac{\hat{t}_i-\bar{t}}{\sigma _i}\bigg )^2,
\end{eqnarray*}

where $d\!f$ is the number of ‘degrees of freedom’ ($d\!f=n-1$). Note that, if $\sigma _i=1$ for all *i* then equation (2) is identical to the sample variance. Scenario 1 is rejected in favour of Scenario 2 if $\mbox{MSWD}> 1+2\sqrt{2/d\!f}$ (at a 95% confidence level, provided that $d\!f> 3$ [2]). Importantly, low ($\mbox{MSWD}< 1+2\sqrt{2/d\!f}$) values do *not* mean that Scenario 1 is *accepted*. In statistics, as in science in general, hypotheses cannot be proved. They can only be disproved.

There are four possible outcomes for the MSWD test: Scenario 1 may be true or false, and the MSWD may be below or above the cutoff. There are two ways to make a correct decision, and two ways to make a mistake. The mistakes are known as type-I and type-II errors.

- Type-I errors (false positives) are committed when Scenario 1 is true, but the MSWD exceeds the critical value. Their probability of occurrence is usually referred to as $\alpha$. If the $1+2\sqrt{2/d\!f}$ MSWD cutoff is used then $\alpha \approx {0.05}$.
- Type-II errors (false negatives) are committed when Scenario 1 is false, but the MSWD is below the critical value. Their probability of occurrence is referred to as $\beta$. The probability $1-\beta$ is also known as the *power* of a statistical test [3].

**Figure 1.**
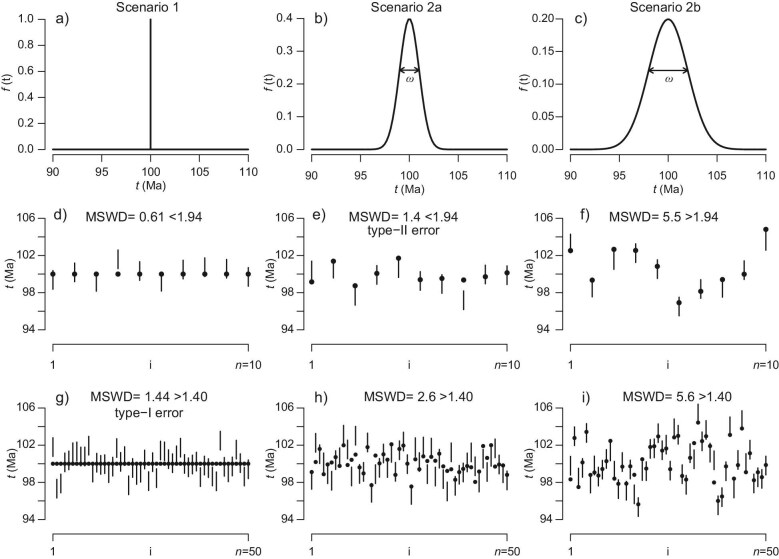
(a–c) Three hypothetical zircon age distributions, with dispersion parameters $\omega = 0$, 1 and 2 Ma, respectively; (d–f) synthetic samples from the three distributions, containing $n=10$ dates each (MSWD cutoff $=1.94$); and (g–i) three further samples with $n=50$ dates each (MSWD cutoff $=1.40$). True ages are shown as black circles; measured dates and their uncertainties are shown as vertical error bars (shown at $1\sigma$). Comparison of the MSWD values with the $1+2\sqrt{2/(n-1)}$ cutoff leads to the correct decision in all cases except for panel (e) (type-II error) and panel (g) (type-I error).

Figure 1 illustrates these important concepts using six synthetic datasets from three idealised hypothetical samples.

The first row of Fig. 1 (panels (a–c) shows the true age distributions of the three samples. The first sample (panel a) follows Scenario 1 with $t=100$ Ma. The second sample (panel b) follows Scenario 2 for a normal age distribution with mean $\mu =100$ Ma and standard deviation $\omega =1$ Ma. Finally, the third sample (panel c) follows Scenario 2 for a normal age distribution with mean $\mu =100$ Ma and standard deviation $\omega =2$ Ma.

The second row of Fig. 1 (panels d–f) shows three random samples of $n=10$ dates. For the sake of simplicity, these dates have a fixed analytical uncertainty of 1 Ma (i.e. $\sigma _i=\sigma =1$ Ma for all *i*). The third row of Fig. 1 (panels g–i) shows another three samples from the same distributions and with the same analytical uncertainty, but a larger sample size ($n=50$). The two sample sizes correspond to slightly different MSWD cutoffs of 1.94 (for $n=10$) and 1.40 (for $n=50$), respectively.

Comparison of the observed MSWDs with these cutoffs results in six decisions. Two of these decisions are wrong. Figure 1g rejects the true Scenario 1, corresponding to a type-I error, whereas Fig. 1e fails to reject the false Scenario 1, corresponding to a type-II error. The probability of a type-I error is fixed at $\alpha =0.05$. In contrast, the probability of a type-II error depends on two factors.

The first factor that affects the power of the MSWD test is the magnitude of the difference between Scenarios 1 and 2. This is illustrated by comparison of Scenarios 2a and 2b. Scenario 1 ($\omega =0$ Ma) is more different from Scenario 2b ($\omega =2$ Ma) than it is from Scenario 2a ($\omega =1$ Ma). Consequently, Scenario 1 is more easily rejected for datasets drawn from population 2b than from population 2a, and the probability of committing a type-II error is correspondingly lower. For a sample size of $n=10$, it can be shown that $\beta =0.54$ for Scenario 2a (Fig. 1f) and $\beta =0.059$ for Scenario 2b (Fig. 1i). In other words, the excess dispersion is 54% likely to remain undetected for Scenario 2a, but only 5.9% for Scenario 2b.

The second factor affecting $\beta$ is sample size (*n*): the larger the sample, the easier it is to detect departures from Scenario 1. Recall that, under Scenario 2a, the probability of committing a type-II error is 54% if $n=10$. This drops to 5.6% ($\beta =0.0056$) if $n=50$ (Fig. 1f). In other words, a five-fold increase in sample size increases the power to reject Scenario 1 by nearly a factor of ten. A similar increase in power is found for Scenario 2b, where the risk of incurring a type-II error reduces from $\beta =0.058$ for $n=10$ to a negligible $\beta =1.5\times {10}^{-7}$ for $n=50$.

The strong dependence of statistical power on sample size means that, given a large enough sample, even the tiniest departure from Scenario 1 becomes detectable. This observation is important because there is a widespread misconception in geochronology that Scenario 1 is desirable and Scenario 2 is not. In reality, the opposite is true. Scenario 1 assumes that all zircons crystallise at exactly the same time (i.e. within the same nanosecond). This is clearly unrealistic. In real geological settings, zircon crystallisation always spans a finite range of time, as under Scenario 2. Given a large enough sample or precise enough data, this time span eventually becomes detectable, resulting in an ‘unacceptably’ high MSWD.

Some have advocated the use of MSWDs as a screening tool to decide whether dates can be averaged or not [4]. In the context of isochron regression, high-MSWD fits are commonly referred to as ‘errorchrons’ [5], an undeniably pejorative term. The author is aware of instances where high MSWD datasets were deemed to be ‘unpublishable’ by reviewers. High MSWD datasets are virtually absent from high-impact publications. These negative connotations may tempt some scientists to ‘cherry pick’ data, by removing perceived ‘outliers’ until the MSWD is reduced to an ‘acceptable’ level. Cherry picking is harmful to science. It increases the occurrence of type-II errors, degrades the reproducibility of experiments, and discards potentially valuable geological information.

The purpose of this paper is to provide the reader with a counter-argument against knee-jerk rejections of high MSWD datasets. Dispersion should not be removed but quantified. Under Scenario 2 of our synthetic zircon example, this means estimating the dispersion parameter $\omega$. For very high MSWD values (${\gt}20$, say), doing so can be as simple as taking the standard deviation of the dates. For lower MSWD values ($1+2\sqrt{2/d\!f}< \mbox{MSWD}< 20$), quantifying the dispersion requires more sophisticated random effect models [6] or Bayesian inversion methods [7]. These concepts do not only apply to weighted means, but can also be generalised to isochron regression [8].

In the context of zircon dating, the dispersion of U–Pb dates may quantify the longevity of magma systems [9]. In thermochronology, it can be used to estimate the cooling rate of exhuming rocks [6]. Steady improvements in analytical precision and throughput across all geochronological techniques mean that overdispersed datasets will become even more prevalent in the future. Our ability to quantify dispersion improves in tandem with these developments, opening new areas of research.

In conclusion, high MSWD datasets are the rule, not the exception. Publications containing consistently low MSWD datasets should not be celebrated but approached with a healthy dose of scepticism.
